# Changes in the End-of-Life Process in Patients with Life-Limiting Diseases through the Intervention of the Pediatric Palliative Care Team

**DOI:** 10.3390/jcm12206588

**Published:** 2023-10-18

**Authors:** Jung Eun Kwon, Yeo Hyang Kim

**Affiliations:** Department of Pediatrics, School of Medicine, Kyungpook National University, Pediatric Palliative Care Center, Kyungpook National University Children’s Hospital, Daegu 41404, Republic of Korea; lovecello623@gmail.com

**Keywords:** advanced care planning, child, intubation, palliative care

## Abstract

Kyungpook National University Children’s Hospital initiated pediatric palliative care (PPC) services in January 2019, focusing on children and adolescents with life-limiting conditions (LLC). A study examined changes in the end-of-life processes in patients with LLC before and after a PPC intervention. This study included 48 deceased patients under 18 years at the hospital, divided into two groups: January 2015 to December 2016 without PPC (25 patients, Period 1) and January 2019 to April 2022 with PPC (23 patients, Period 2). Analysis of medical records revealed the following: no age/sex differences; more active advanced care planning in Period 2 (15/23 vs. 7/25, *p* = 0.01); discussing withholding/withdrawing treatment increased in Period 2 (91.3% vs. 64.0%, *p* = 0.025); intubation and CPR were less frequent in Period 2 (intubation 2/23 vs. 19/25, *p* = 0.000; CPR 3/23 vs. 11/25, *p* = 0.018); Period 1 had more deaths in the ICU (18/25 vs. 10/23, *p* = 0.045); and 3 patients in Period 2 chose home deaths. A survey in Period 2 revealed high satisfaction with emotional support (91.7%), practical assistance (91.6%), and symptom management (83.3%). PPC facilitated discussions on advanced care planning and treatment choices, ensuring peaceful and prepared farewells for children with LLC and their families.

## 1. Introduction

Pediatric palliative care (PPC) is provided for babies, children, and young people with incurable life-limiting conditions (LLC) that can lead to premature death, as well as for those with life-threatening conditions that may be curable but can still fail to respond to treatment [1].

Due to the advances in medical science, people are now living longer, resulting in an increased survival rate among pediatric patients with LLC. However, some diseases remain incurable, leading to unavoidable premature death. A previous study on the survival rate within one year after discharge from the pediatric intensive care unit (PICU) found that 99% of patients without LLC survived up to a year after discharge, whereas patients with LLC had a significantly lower survival rate of up to three months after PICU discharge. Moreover, patients with LLC were 2.5 times more likely to die compared with those without LLC [2].

Therefore, it is essential to have effective communication between patients, guardians, and medical staff at the time of LLC diagnosis, during the treatment process, or when the disease worsens (and death becomes imminent). This includes establishing advanced care planning and making decisions regarding life-sustaining treatment. Advanced care planning involves discussions between families and medical professionals to set treatment and care goals for children and adolescents. This process involves providing information about the current and expected health conditions, including prognosis, as well as explaining the potential benefits and drawbacks of various treatment options [3]. This process helps patients and their families to make informed choices about their care [4].

In Korea, even if advanced care planning cannot be established, the “Act on Decisions on Life-sustaining Treatment for Patients in Hospice and Palliative Care or at the End of Life”, enforced in February 2018, allows doctors and parents to make life-sustaining treatment decisions for pediatric patients with LLC. Under the current law, these decisions for children are limited to end-of-life situations. However, expanding the scope of these decisions to encompass children would be meaningful as it could reduce futile life-sustaining treatment and aid in preparing patients and their families for the end-of-life period.

Since July 2018, a pilot project for PPC, a palliative care service for LLC patients and their families, has been implemented in Korea. This project includes services such as physical and psychosocial support, assistance in the decision-making process, end-of-life care, and support for bereaved families.

The palliative approach for LLC patients and their families, including the establishment of advanced care planning, the enforcement of the Act on Life-sustaining Treatment Decisions, and the PPC pilot project, appears to have impacted the end-of-life processes in LLC patients. This study aims to assess changes in the end-of-life processes in patients with LLC with a PPC team intervention and to evaluate the satisfaction of bereaved families with the team’s intervention.

## 2. Materials and Methods

### 2.1. Participants and Survey

Forty-eight patients under the age of eighteen who passed away at Kyungpook National University Children’s Hospital due to life-limiting conditions (LLC) were included in this study. Starting from January 2019, when pediatric palliative care (PPC) services commenced, patients were categorized into two groups based on the availability of PPC services: (1) those who died between January 2015 and December 2016 without access to PPC services (Period 1), and (2) those who died between January 2019 and April 2022 with PPC services (Period 2).

Patients’ clinical information was retrospectively gathered from their medical records, including age, gender, establishment of advanced care planning, decisions about withholding or withdrawing life-sustaining treatment, endotracheal intubation, cardiopulmonary resuscitation (CPR) during the end-of-life phase, and the place of death.

A survey on treatment and care service satisfaction (FAMCARE-2) [5] was administered to families who experienced bereavement during Period 2, aiming to assess the adequacy of patient and family care.

### 2.2. Definitions

Definitions for all terminologies were sourced from the “Korean Professional Consensus for Comfort Care and Withdrawing/Withholding in the Intensive Care Unit” by the Task Force of the Korean Society of Critical Care Medicine [6] and the “Act on Decisions on Life-sustaining Treatment for Patients in Hospice and Palliative Care or at the End of Life” [7].

LLC refers to a condition that is expected to result in a child’s death before reaching adulthood, thus shortening life expectancy [4].

The establishment of advanced care planning refers to the comprehensive process of discussing care goals and intentions for medical treatment in the present and during disease exacerbation, involving medical staff and the family [4].

Life-sustaining treatment encompasses CPR, hemodialysis, administration of anticancer drugs, ventilator treatment, vasopressor use, and other medical procedures stipulated by the Presidential Decree, which prolong the end-of-life phase without a curative effect. Additionally, general treatment provided in the intensive care unit may fall under this category [6,7].

The end-of-life phase signifies a state in which survival is not possible, recovery is unattainable despite treatment, symptoms deteriorate rapidly, and death is imminent [7].

### 2.3. Statistical Analysis

All statistical analyses were executed using IBM SPSS Statistics for Windows, version 26.0 (IBM Co., Armonk, NY, USA). Descriptive statistical analyses were performed on patients’ demographic characteristics. Chi-squared tests were utilized to discern differences between the two periods. Significance was set at *p* < 0.05.

## 3. Results

### 3.1. Patients’ General Characteristics

Table 1 presents the general and clinical attributes of the patients in this study. A total of 48 patients with life-limiting conditions (LLC) who passed away between January 2015 and April 2022 were included. The patient count per period was 25 in Period 1 and 23 in Period 2.

Among the patients, 56.2% were male and 43.8% were female. The age group most common at the time of death was 1–9 years old, comprising 50% of the cases. No significant differences were observed in gender or median age at the time of death (6 years, ranging from 0 to 19 years) between Periods 1 and 2. Neurological disease (64.0%) was the predominant underlying condition in Period 1, while haemato-oncological disease (39.1%) took precedence in Period 2.

### 3.2. Support in the Decision-Making Process

Table 2 displays the analysis results concerning support for the decision-making process among patients and their families. In Period 1, advanced care planning was discussed or established for 28% of patients, while this figure rose to 65.2% in Period 2 (*p* = 0.01). The active discussion and implementation of decisions regarding the withdrawal or withholding of life-sustaining treatment were more pronounced in Period 2 (91.3%) compared to Period 1 (64%) (*p* = 0.025).

During the end-of-life phase, endotracheal intubation was administered in 76% of deaths during Period 1, but only in 0.8% of deaths during Period 2 (*p* = 0.000). Furthermore, CPR was conducted in 44% of deaths during Period 1 and 13% of deaths during Period 2 (*p* = 0.018).

### 3.3. Change in Place of Death

**Figure 1:**
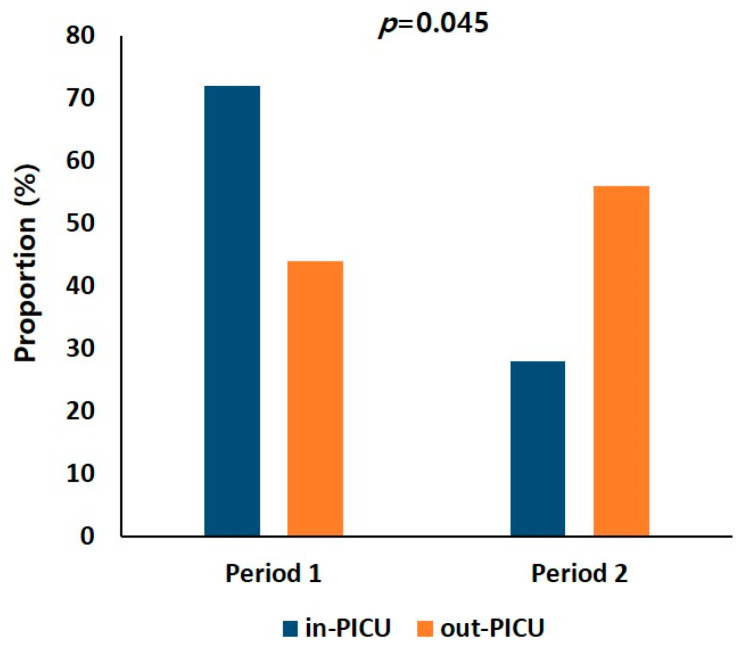
Changes in the location in the end-of-life care of patients. After the intervention of the pediatric palliative care team, the proportion of end-of-life care outside the intensive care unit increased significantly.

Figure 1 illustrates the distribution of patients’ locations of death. In Period 1, a majority of deaths occurred in the intensive care unit (72%). However, in Period 2, there was a notable increase (56.5%) in deaths outside the intensive care unit, including home deaths (*p* = 0.045).

During Period 2, three families opted for their loved ones’ passing to take place at home. Of these cases, two patients had brainstem glioma, and one patient had hypoxic-ischemic encephalopathy along with multiple organ failure.

### 3.4. Service Satisfaction Survey of Bereaved Families

Table 3 presents the outcomes of the satisfaction survey conducted among bereaved families during Period 2, using the FAMCARE-2 tool. Among the total patient families, twelve families (52%) responded, and the majority of responses indicated that families were “very satisfied/satisfied” across all questions. Families expressed contentment with the emotional support provided to both the patient and their family (91.7%), practical assistance from the PPC team (91.6%), and the PPC team’s adept management of the patient’s physical symptoms (83.3%). Particularly, for seven items (no. 1, 4, 9, 10, 11, 16, and 17), over 90% of bereaved families responded that they were “very satisfied/satisfied.” In relation to item no. 9, “Availability of the palliative care team to the family”, all respondents (100%) indicated being “very satisfied/satisfied”.

## 4. Discussion

This study demonstrates that the involvement of the PPC team for pediatric LLC patients and their families helps to ensure that both patients and families receive greater respect during the end-of-life journey, enabling them to spend more meaningful time together.

Discussing the magnitude of LLCs and the death of pediatric patients poses challenges for medical staff, patients, and families alike. Owing to these challenges are the establishment of advanced care planning and decisions about life-sustaining treatment that predominantly transpire when a child’s death is imminent [8]. Such unprepared deaths among children inflict deeper sorrow upon families and prolong the mourning process [9]. Thus, the discourse surrounding advanced care planning and life-sustaining treatment becomes a pivotal and essential process. Ideally, the PPC team assesses LLC patients via initial counseling, subsequently creating a comprehensive care plan [10]. This study found that the PPC team, armed with extensive expertise in caring for pediatric patients with LLC, actively guided patients and their families through the participation in decision-making processes in the treatment and care of patients, which was facilitated by systems such as advanced care planning and life-sustaining treatment choices.

In the United States and Europe, the rate of withdrawing or withholding life-sustaining treatment for terminally ill children and adolescents ranges from 20% to 55% [11,12,13,14]. In Korea, decisions concerning CPR or its cessation were often made during consultations with patients’ families only when death was imminent. However, recent practices involve abstaining from or discontinuing medical procedures that extend the end-of-life process without treatment effects, such as CPR, intubation, hemodialysis, and vasopressor use, when a patient faces a bleak prognosis and is undergoing end-of-life processes. In this study, the percentages of decisions to withhold life-sustaining treatment without endotracheal intubation and CPR during the end-of-life phase escalated from 24% and 56% (in the past) to 91% and 87%, respectively.

As per the Korean Life-Sustaining Treatment Decision Act, determinations regarding life-sustaining treatment for children and adolescents are confined to the end-of-life period. Such decisions are based on medical practitioners’ assessments and the family’s verdict, particularly that of the parents. Consequently, interactions with medical staff and participation in decision-making processes impact families’ lives post-bereavement [9]. A prior study involving parents bereaved by childhood cancer revealed that active consultation with the PPC medical staff before a child’s death positively influenced the mourning process afterward [15]. This outcome signifies that patients and families were well-informed and garnered comprehensive support from the PPC team. Consistent with this prior study, this research also highlighted that patients’ and families’ preferences were integrated into the care plan, facilitating well-informed decisions through high-quality communication with the PPC team.

Korean pediatric death statistics indicate that 85.5% of children with LLC pass away in hospitals [16]. Moreover, they are more prone to die within the intensive care unit compared to their counterparts without LLC [2]. However, this study, where the PPC team was involved in the end-of-life care, reveals that the majority of the end-of-life events in LLC patients occurred in general wards rather than within intensive care units. A special note was warranted for the three families who opted for home as the place of death. The PPC team offered continuous symptom management via consultation, imparted end-of-life education, and provided direct care during patients’ final moments. Additionally, for families selecting home as the place of death, the team extended home visits and offered a 24 h emergency call service through home care programs, thus enabling patients to spend their final moments in a comfortable familial environment.

In a previous study regarding the preferred place of death for pediatric patients, parents, children, and service providers, as well as the outcomes of bereavement, a preference for home deaths was uniformly expressed [17]. Furthermore, parents’ strategic planning for their child’s place of death led to increased decisions favoring home deaths, where preparations for the child’s passing were undertaken in advance, engendering a sense of comfort [18]. Among the types of hospice palliative care in Korea, home-type hospices exist, albeit with disease-specific limitations. This study underscores that the availability of adequate resources and support from the PPC team empowers patients and their families to opt for comfortable locations, be it their home or a private room within a general ward.

The PPC team’s approach to decision making and end-of-life care embodies a “family-centered care” model. This approach is grounded in collaborative interactions between medical professionals, patients, and families, fostering improved healthcare outcomes and heightened satisfaction among patients and families. By collaboratively sharing information about children and devising treatment plans that cater to both the child’s and parent’s needs, medical staff and parents collectively steer the course of care [19]. The outcomes of the service satisfaction survey conducted for bereaved families in this study underscores their contentment with family participation in decision making, accessibility to the PPC team, symptom management, and emotional support provided to patients and their families. This signifies that the PPC team’s involvement in patients’ end-of-life processes was fitting. This family-centered approach in PPC endeavors to actualize the overarching goals of PPC—ensuring that patients’ choices are met and enhancing the quality of life for both patients and families. Notably, PPC is anticipated to contribute to post-traumatic growth rather than trauma, particularly concerning the death of an ailing child, for parents and siblings.

This study’s limitations include its retrospective design, which relied on medical records, thereby limiting the incorporation of diverse factors that may have influenced changes in the end-of-life process of pediatric patients across different periods. Further mixed-method studies incorporating both quantitative and qualitative approaches are warranted to provide a more comprehensive understanding. Moreover, due to its single-institution scope, caution is needed when attempting to generalize the findings as a universal pattern applicable to all institutions offering pediatric palliative care.

## 5. Conclusions

In conclusion, the PPC team plays a crucial role by offering information and consultation avenues for advanced care planning and life-sustaining treatment choices. This enables pediatric patients and their families dealing with LLC to experience a serene passing and a well-prepared farewell.

## Figures and Tables

**Table 1:** General and clinical characteristics of the patients.

Characteristics	Total	Period 1 (*n* = 25)	Period 2 (*n* = 23)
Sex			
Male	27 (56.2)	16 (64.0)	11 (47.8)
Female	21 (43.8)	9 (36.0)	12 (52.2)
Age (years)			
<1	5 (10.4)	3 (12.0)	2 (8.7)
1–9	24 (50)	12 (48.0)	12 (52.2)
10–19	19 (39.6)	10 (40.0)	9 (39.1)
Main problem			
Neurology	23 (47.9)	16 (64.0)	7 (30.4)
Haemato-oncology	15 (31.2)	6 (24.0)	9 (39.1)
Cardiology	6 (12.5)	0 (0.0)	6 (26.1)
Pulmonology	2 (4.2)	2 (8.0)	0 (0.0)
Gastroenterology	2 (4.2)	1 (4.0)	1 (4.3)

Values are presented as numbers (%). Abbreviation: *n*, number.

**Table 2:** The comparison of end-of-life decisions of the patients according to the pediatric palliative care service.

Valuables	Period 1 (*n* = 25)		Period 2 (*n* = 23)		*p*
	Yes	No	Yes	No	
Advanced care planning	7 (28.0)	18 (72.0)	15 (65.2)	8 (34.8)	0.01
Life-sustaining treatment	9 (36.0)	16 (64.0)	2 (8.7)	21 (91.3)	0.025
End-of-life process					
Intubation	19 (76.0)	6 (24.0)	2 (8.7)	21 (91.3)	0.000
CPR	11 (44.0)	14 (56.0)	3 (13.0)	20 (87.0)	0.018

Values are presented as numbers (%). Abbreviation: *n*, number; CPR, cardiopulmonary resuscitation.

**Table 3:** Responses to each of the FAMCARE-2 items [5].

Item Description	Response (%)		
	Very Satisfied/ Satisfied	Neither	Dissatisfied/Very Dissatisfied
1. The patient’s comfort	91.7	8.3	0.0
2. The way in which the patient’s condition and likely progress were explained by the palliative care team	83.3	8.3	8.3
3. Information given about the side effects of treatment	75.0	25.0	0.0
4. The way in which the palliative care team respects the patient’s dignity	91.7	8.3	0.0
5. Meetings with the palliative care team to discuss the patient’s condition and plan of care	83.3	16.7	0.0
6. Speed with which symptoms are treated	75.0	25.0	0.0
7. Palliative care team’s attention to the patient’s description of symptoms	83.4	16.7	0.0
8. The way in which the patient’s physical needs for comfort are met	83.3	16.7	0.0
9. Availability of the palliative care team to the family	100	0.0	0.0
10. Emotional support provided to family members by the palliative care team	91.6	8.3	0.0
11. The practical assistance provided by the palliative care team (e.g., bathing, home care, respite)	91.6	8.3	0.0
12. The doctor’s attention to the patient’s symptoms	83.3	16.7	0.0
13. The way in which the family is included in treatment and care decisions	83.4	16.7	0.0
14. Information given about how to manage the patient’s symptoms (e.g., pain, constipation)	83.4	8.3	8.3
15. How effectively the palliative care team manages the patient’s symptoms	83.3	16.7	0.0
16. The palliative care team’s response to changes in the patient’s care needs	91.7	8.3	0.0
17. Emotional support provided to the patient by the palliative care team	91.7	8.3	0.0

## Data Availability

The data presented in this study are available upon request from the corresponding author.

## References

[1] Chambers L. (2018). A Guide to Children’s Palliative Care; Supporting Babies, Children and Young People with Life-Limiting and Life-Threatening Conditions and Their Families.

[2] Fraser L.K., Parslow R. (2018). Children with life-limiting conditions in paediatric intensive care units: A national cohort, data linkage study. Arch. Dis. Child..

[3] Spicer S., Macdonald M.E., Davies D., Vadeboncoeur C., Siden H. (2015). Introducing a lexicon of terms for paediatric palliative care. Paediatr. Child. Health.

[4] Shin H.Y., Kim M.S., Kang S.H., Kim C.H., Moon Y.J., Song I.G. (2019). A Practical Guide for Pediatric Advance Care Planning.

[5] Aoun S., Bird S., Kristjanson L.J., Currow D. (2010). Reliability testing of the FAMCARE-2 scale: Measuring family carer satisfaction with palliative care. Palliat. Med..

[6] Ko S.B., Kim Y.J., Kim J.H., Ra S.H., Moon J.Y., Park S.Y. (2018). Korean Professional Consensus for Comfort Care and Withdrawing/Withholding in the Intensive Care Unit.

[7] Act on Decisions on Life-Sustaining Treatment for Patients in Hospice and Palliative Care or at the End of Life. https://law.go.kr.

[8] Durall A., Zurakowski D., Wolfe J. (2012). Barriers to conducting advance care discussions for children with life-threatening conditions. Pediatrics.

[9] Van der Geest I.M., Darlington A.S.E., Streng I.C., Michiels E.M., Pieters R., van den Heuvel-Eibrink M.M. (2014). Parents’ experiences of pediatric palliative care and the impact on long-term parental grief. J. Pain Symptom Manag..

[10] The Guideline for Korea Pediatric Palliative Care Project. https://hospice.go.kr.

[11] Burns J.P., Mitchell C., Outwater K.M., Geller M., Griffith J.L., Todres I.D., Truog R.D. (2000). End-of-life care in the pediatric intensive care unit after the forgoing of life-sustaining treatment. Crit. Care Med..

[12] Garros D., Rosychuk R.J., Cox P.N. (2003). Circumstances surrounding end of life in a pediatric intensive care unit. Pediatrics.

[13] Althabe M., Cardigni G., Vassallo J.C., Allende D., Berrueta M., Codermatz M., Córdoba J., Castellano S., Jabornisky R., Marrone Y. (2003). Dying in the intensive care unit: Collaborative multicenter study about forgoing life-sustaining treatment in Argentine pediatric intensive care units. Pediatr. Crit. Care Med..

[14] Devictor D.J., Nguyen D.T. (2004). Forgoing life-sustaining treatments in children: A comparison between Northern and Southern European pediatric intensive care units. Pediatr. Crit. Care Med..

[15] Kreicbergs U.C., Lannen P., Onelov E., Wolfe J. (2007). Parental grief after losing a child to cancer: Impact of professional and social support on long-term outcomes. J. Clin. Oncol..

[16] Kim M.S., Lim N.G., Kim H.J., Kim C., Lee J.Y. (2018). Pediatric deaths attributed to complex chronic conditions over 10 years in Korea: Evidence for the need to provide pediatric palliative care. J. Korean Med. Sci..

[17] Johnston E.E., Martinez I., Currie E., Brock K.E., Wolfe J. (2020). Hospital or home?. Where should children die and how do we make that a reality? J. Pain Symptom Manag..

[18] Dussel V., Kreicbergs U., Hilden J.M., Watterson J., Moore C., Turner B.G., Weeks J.C., Wolfe J. (2009). Looking beyond where children die: Determinants and effects of planning a child’s location of death. J. Pain Symptom Manag..

[19] Shields L., Pratt J., Davis L., Hunter J. (2007). Family-centred care for children in hospital. Cochrane Database Syst. Rev..

